# Dispersal and life-history traits in a spider with rapid range expansion

**DOI:** 10.1186/s40462-019-0182-4

**Published:** 2020-01-07

**Authors:** Marina Wolz, Michael Klockmann, Torben Schmitz, Stano Pekár, Dries Bonte, Gabriele Uhl

**Affiliations:** 1 Zoological Institute and Museum, General and Systematic Zoology, University of Greifswald, Greifswald, Germany; 2 Masaryk University, Brno, Czech Republic; 3 Ghent University, Ghent, Belgium

**Keywords:** Reciprocal common garden experiment, Passive dispersal, Body size, Reproductive success, *Argiope bruennichi*, Araneae

## Abstract

**Background:**

Dispersal and reproduction are key life-history traits that jointly determine species’ potential to expand their distribution, for instance in light of ongoing climate change. These life-history traits are known to be under selection by changing local environmental conditions, but they may also evolve by spatial sorting. While local natural selection and spatial sorting are mainly studied in model organisms, we do not know the degree to which these processes are relevant in the wild, despite their importance to a comprehensive understanding of species’ resistance and tolerance to climate change.

**Methods:**

The wasp spider *Argiope bruennichi* has undergone a natural range expansion - from the Mediterranean to Northern Europe during the recent decades. Using reciprocal common garden experiments in the laboratory, we studied differences in crucial traits between replicated core (Southern France) and edge (Baltic States) populations. We tested theoretical predictions of enhanced dispersal (ballooning behaviour) and reproductive performance (fecundity and winter survival) at the expansion front due to spatial sorting and local environmental conditions.

**Results:**

Dispersal rates were not consistently higher at the northern expansion front, but were impacted by the overwintering climatic conditions experienced, such that dispersal was higher when spiderlings had experienced winter conditions as occur in their region. Hatching success and winter survival were lower at the range border. In agreement with theoretical predictions, spiders from the northern leading edge invested more in reproduction for their given body size.

**Conclusions:**

We found no evidence for spatial sorting leading to higher dispersal in northern range edge populations of *A. bruennichi*. However, reproductive investment and overwintering survival between core and edge populations differed. These life-history traits that directly affect species’ expansion rates seem to have diverged during the recent range expansion of *A. bruennichi*. We discuss the observed changes with respect to the species’ natural history and the ecological drivers associated with range expansion to northern latitudes.

## Background

Life-history traits such as birth, survival and reproductive traits shape an organism’s life cycle and have a direct impact on fitness. These traits can evolve across a species’ range [1, 2] according to the scale and magnitude of environmental gradients [3, 4]. Along latitudinal gradients, for example, local adaptation of life-history traits in relation to climate and seasonality has been described in many species (e.g. [5–9]). Species ranges are, however, highly dynamic; the most recent decades have witnessed a trend of shifting or expanding ranges towards higher latitudes and altitudes due to rapid climate change [10]. The population dynamics of newly established populations at range edges are typically characterised by extinction and recolonization events [11–13], and are obviously impacted by trait evolution during the expansion dynamics [14, 15]. Species also approach their ecological limit at these borders, where death rates are close to or even exceed birth rates [11, 16]. Populations at the range edge are therefore close to ecological sinks and expected to be smaller, to show a lower resilience and thus to be more prone to extinction under environmental fluctuations [13]. They typically show reduced genetic diversity due to the effects of genetic drift [11].

Under such non-equilibrium conditions described above, the environment does not necessarily impose selection on traits, but trait evolution may primarily result from the underlying spatial dynamics [17]. Spatial sorting, the accumulation of dispersive genotypes, may occur at the leading edges of expansions [17, 18]. If any component of dispersal is heritable, then the offspring of these individuals will have on average higher dispersal ability compared to those from the core of the species distribution, leading to an “Olympic Village effect” [17, 19]. Repeated spatial assortment for dispersal further increases the rates of dispersal on the advancing front and can even result in accelerated range expansion or shifts in expansion dynamics (e.g., [20, 21]). This non-random organisation of phenotypes at the expanding edge is well understood theoretically (reviewed in [14]). Support from empirical studies is also accumulating (reviewed in [18]). However, species that are prone to Allee-effects may be less likely to show spatial sorting, as expansion bouts may promptly go extinct due to low population densities [22, 23]. In addition, low population densities and reduced competition can locally select for *r*-strategies where investment in reproduction is evolutionarily more advantageous than investment in endurance and longevity [3, 19, 24]. In addition, fragmented habitat structure at expansion edges can select against dispersal [15, 16]. At the core of species’ distributions where suitable habitats tend to be abundant, populations are likely density regulated and at their carrying capacity [3, 24]. Spatial sorting and local natural selection are thus strong determinants of contemporary evolution at expansion fronts, and genetic divergence along range gradients will be determined by their joint action [17, 25, 26].

If the processes of spatial sorting and local natural selection are relevant as drivers of life-histories, then signatures should be visible in natural populations. To date, such insights are mostly derived from correlative phenotypic approaches (e.g., [27]), but recently some studies have shown phenotypic divergence to be genetically determined as well (e.g. [28]). As outlined above, irrespective of the exact underlying processes, life-histories at expansion fronts should be characterised by higher dispersal abilities and high reproductive rates when spatial sorting acts in concert with local natural selection in response to population demography. Most insights into these processes stem from either theoretical studies (reviewed in [11, 14, 18, 29]), experimental work using insect model organisms [20], or studies on some iconic species such as the cane toad (reviewed in [17, 19]). However, profound insights on their prevalence in nature are lacking. While dispersal needs to be considered as a central trait in life-history [30, 31], it has been phenotypically studied using proxies such as leg lengths, movement efficiency in animals or seed dispersal structures in plants [20, 28, 32]. Most often, these studies ignore the fact that dispersal is always conditional on environmental conditions experienced during early life in the case of breeding dispersal, or on environmental conditions experienced by mothers in species in which juveniles shown natal dispersal (e.g., maternal effects on dispersal [33–36];). Hence, reaction norms towards environmental drivers of dispersal evolve in response to costs and benefits, such that dispersal rates may not be constant over time (e.g., [37]). In the case of natal dispersal, dispersal will be subject to tension and synergy between parent and offspring fitness maximisation, and thus mediated by the reliability and predictability of the cues which condition dispersal across generations [38–40]. Spiders display a unique dispersal behaviour, which can be studied in controlled conditions in the lab [41]; they drift with air currents during a behaviour called “ballooning” after displaying unique behaviours prior to the dispersal event. Ballooning activity depends on environmental conditions such as temperature and wind velocity [42, 43] and on the body size of the disperser; offspring disperse after hatching in larger species, whereas adult stages may balloon in small species.

In this study, we assessed dispersal behaviour and its integration into life-histories between core and edge populations of the large orb-weaving spider *Argiope bruennichi*. This species has been expanding its range considerably from the Mediterranean northwards into the Baltic States, Scandinavia and UK during the last decades [44, 45]. Juvenile stages (spiderlings) of the orb-web spider engage in passive dispersal after their emergence from egg sacs in spring by releasing silk threads for ballooning. We subjected egg sacs containing hatched spiderlings to one of two climatic conditions during the overwintering phase in a reciprocal common garden setting. We tested the prediction of increased dispersal in northern edge populations from the Baltic States relative to populations from the core in Southern France. To determine whether changes in dispersal are due to spatial sorting or the climatic conditions experienced prior to hatching, we investigated dispersal activity in spiderlings who experienced either of two winter regimes (conditions matching their source location or non-matching). To obtain a broader overview of the importance of winter conditions on life-histories, and putative correlations with dispersal, we investigated the reproductive output and differential survival of spiderlings from core and northern edge populations.

## Methods

### The study species

The Palearctic orb-weaving spider *Argiope bruennichi* (Scopoli, 1772) (Araneidae) has undergone a substantial range expansion in Europe. From a Mediterranean distribution, it has expanded northwards and can now be found in Estonia, Sweden and recently in Finland. Records of single individuals of *A. bruennichi* have been reported in the Baltic States in 2002 for Lithuania [46], and in 2009 for Latvia (https://dabasdati.lv/en) and the southwest of Estonia (https://elurikkus.ee; http://loodus.keskkonnainfo.ee). A handful of spiders were reported from different locations in southern Finland following a public survey in 2013 (http://biolcoll.utu.fi/arach/aran2013/aranmaps.htm). Apart from these database records, further information on the species’ range expansion is compiled in [44, 45, 47–49].

Females are 14–17 mm in body size and are much larger than the males [49]. After mating in summer, females continue to forage and eventually oviposit ca. 200 eggs into an intricately woven egg sac [50] before dying in autumn [51]. The spiderlings hatch from the eggs (as postembryos) about 2 weeks after oviposition [52]. Postembryos moult once to first instar spiderlings in the egg sac and emerge from it during the following spring [49]. Ballooning behaviour, if it occurs, is restricted to the juvenile stages of this species [51, 53]. After several moults the spiders reach maturity in summer.

### Sampling & life-history data

**Fig. 1:**
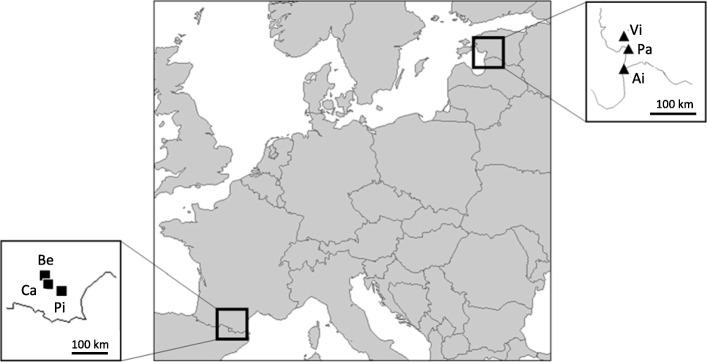
Sampling sites for adult female *Argiope bruennichi*. Black frames mark the regions in Southern France and the Baltic States (Estonia and Latvia) from which spiders were collected. Black squares show the sampling locations of females in Southern France (Be = Belflou, Ca = Casties, Pi = Pieusse). Black triangles show the sampling locations in the Baltic States (Vi = Virtsu, Pa = Pärnu in Estonia and Ai = Ainaži in Latvia). The map was generated using RStudio Version 3.3.2 (2016-10-31). Coordinates are given in the supplemental S1

Adult female *A. bruennichi* were collected from the field in 2016. The core region of the distribution is represented by populations from Southern France. Here, we collected adult females during 3 days in August 2016 (18th - 20th) from extensively managed meadows and ditches. Spiders from the edge of the distribution were also collected in August 2016 (20th – 28th) from the Baltic States Latvia and Estonia. At the northern edge, suitable habitat appeared to be more patchily distributed making it difficult to find suitably large populations to sample. Based on previous field experience from 2015 (G. Uhl unpublished), the collection dates in both regions were selected to match the period late in the mating season when it is highly probable that females have mated but not yet laid eggs. The absence of males at the sites (males are cannibalised during mating) and the lack of egg sacs in the field indicated that the chosen sampling dates corresponded to the appropriate period of the species’ life cycle. We chose three populations from the core and three from the edge of the distribution (Fig. 1, Additional file 1: Table S1). In each region, the replicate populations were 20 to 60 km apart. Adult females were taken back to the laboratory and housed in individual containers (1000 ml) where they subsequently laid eggs (France *n* = 82, Baltic States *n* = 96). The females were kept in one of two climate chambers that simulated the climatic conditions for the regions they were sampled from (Southern France or the Baltic States). Climate simulations were built on data that we retrieved from data-loggers (temperature and humidity) that had been placed at the collecting sites in the previous year (2015–2016). Humidity was set to 80% for both chambers. Females were fed regularly with a controlled number of flies (*Calliphora spec.*). After oviposition, females were anesthetized and preserved in ethanol, whilst egg sacs remained in the respective climate chambers to allow the postembryos to hatch under simulated natural conditions. Hatching already occurs after about 2 weeks following oviposition [52]. Following hatching, a laboratory reciprocal common garden experiment (see below) was then conducted by subjecting the 178 egg sacs from the two regions with the postembryos inside to either native or foreign winter conditions (for natural and simulated temperature regimes see supplementary material Additional file 1: Figure S1, Additional file 1: Table S2).

When the common garden winter treatment was completed, we determined clutch size, hatching success and overwintering survival of offspring. Clutch size was taken as the total number of eggs in an egg sac (this included both live and dead spiderlings and unhatched eggs). Hatching success was established as the number of hatched spiderlings (live plus dead) divided by the total number of eggs produced in an egg sac. For all females which produced egg sacs which successfully hatched, the winter survival of offspring was calculated as the number of live spiderlings after the winter treatment divided by the total number of hatched spiderlings (live plus dead). The body size of adult females was recorded (*N* = 113, one data point is missing as one female was not labelled unequivocally). Body size was measured as the total length of patella + tibia (in mm) of the first right leg; a measurement that is highly correlated to prosoma width in this species (Pearson correlation: r = 0.917, *p* < 0.001, *N* = 302) and which is generally used as a proxy for body size in spiders (e.g. [54]). Similarly, clutch size was established for the 114 females whose offspring were subject to the ballooning trial (one data point missing, *N* = 113). Due to one missing data point for female body size and one for clutch size, the analysis of the effect of origin and female body size on clutch size is based on *N* = 112. Hatching success was established for all females (*N* = 178) and survival of offspring after the common garden conditions was established for all egg sacs with hatching success (*N* = 163). Note that, in *A. bruennichi*, offspring do not overwinter as fertilized eggs but as spiderlings that remain in the egg sac until the following spring [51].

We quantified variation in life-history traits under standardized lab conditions and interpret these throughout the manuscript from an evolutionary perspective. While offspring dispersal is assessed in a common environment (with quantified dam-components of the observed variance), changes in ballooning nevertheless may originate from maternal effects (see [35]). Clutch sizes are likely directly impacted by the developmental conditions experienced by females in the field. Consequently, for non-model organisms such as *A. bruennichi*, separating the genetic effects from the environmental and paternal effects is difficult. These effects could be disentangled using laboratory populations raised over several generations. However, efforts to do so in *A. bruennichi* resulted in insufficient numbers of offspring surviving to maturity and from only a subset of families. Lab populations will therefore go through bottlenecks and are subject to genetic drift and the resulting test populations would not represent natural populations. Further, asynchrony in maturation (females are much larger and undergo about twice as many moults as males in this species) is more pronounced in the lab than in the field. It can happen that males mature and die before females reach maturity, which further reduces the number of mating partners to set up the next generation. Severe bottlenecks and non-random mating under laboratory conditions clearly constrain investigations on genetic adaptation and plasticity in non-model species. Although using model species could help to overcome these difficulties, a major drawback would be that the majority of species with interesting distribution dynamics are non-model species and secondly, long-established laboratory stocks of model species may also be subject to the effects of drift if they are established from only a handful of individuals as is often the case.

### Laboratory reciprocal common garden experiment

In order to examine the effects of overwintering in matching or non-matching conditions on spiderling dispersal, we used a fully factorial experimental design in which offspring (hence, spiderlings in egg sacs) from females from southern core and northern edge populations were overwintered under temperature conditions typical for the range core (Southern France) and the edge (Baltic States). Egg sacs were transferred to small plastic containers (5 x 5 x 3.5 cm) in which they were glued to the inner side of the lid. The containers were equipped with gauze-covered windows on two sides to ensure adequate ventilation. At the end of November (23.11.2016), the egg sacs were transferred from the native simulated climate condition to one of two climate cabinets in which the winter conditions of either region was simulated over the course of the winter. The Southern France winter conditions were simulated in a Panasonic MLR-352 and the Baltic winter conditions in a Percival LT-36VL - the latter allowing minus degrees. Frost was generated in the Percival Cabinet by decreasing the temperature to − 2.0 °C during the night for 12 h (freeze-thaw days) or throughout 24 h (ice days). Egg sacs from Southern France winter conditions were transferred to the Percival cabinet to experience the scheduled number of freeze-thaw and ice days (see supplementary material Table S1). This set up also allowed us to detect any differential effects of winter condition on spiderling survival.

Under natural conditions, the postembryos moult once and then emerge in spring from the egg sac as first instar spiderlings [51]. We restricted the ballooning experiments to this moulting stage, as it is the most important dispersal stage (Krüger J. & Uhl G., unpublished). As spiderlings stay in a group for some days after emerging from an egg sac, we mimicked this situation by keeping the spiderlings in a group for 9 days after emergence until the day of the experiment. Starting in late April, we randomly selected five egg sacs per day from each winter treatment, opened them manually, and transferred each opened egg sac with the spiderlings into a larger container (18 × 15.5 × 9 cm) that was likewise equipped with gauze-covered ventilation windows. In these individual “family” containers (*N* = 114), the spiderlings continued to experience the matching native or non-matching foreign temperature treatments. The spiderlings were not fed during this period as under natural conditions they start building webs only after one further moult. The family boxes were sprayed once a day with tap water to guarantee high humidity in the boxes. The general humidity in the climate chambers remained at 80%.

### Dispersal trials

**Fig. 2:**
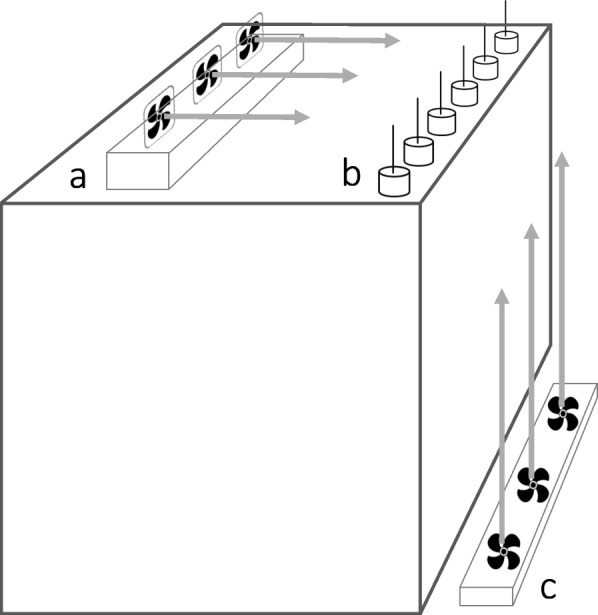
Experimental set -up used for testing ballooning activity in *Argiope bruennichi* spiderlings. Each spiderling was placed individually on a ballooning unit (**b**). Behind the six ballooning units PC ventilators (**a**) were installed on a wooden beam to direct the silk threats. Under the ballooning unit a larger ventilator (**c**) was fixed to generate upward wind velocities. Grey arrows represent the direction of the airflow

A total of 2052 spiderlings from 114 egg sacs that had undergone winter treatment conditions were tested individually for ballooning activity (see supplementary Table S2). We used an experimental set-up consisting of ventilators as described by [55]. Each ballooning unit (Fig. 2) consisted of a vertical wooden skewer (10.4 cm long, 0.15 cm diameter), that was mounted on a platform (plastic film container: 1.8 cm diameter, 5 cm height). Six of these ballooning units were arranged in a line, 3 cm apart, at the front rim of an elevated box (64 x 64 x 54 cm, Fig. 2b). Behind the ballooning units, three PC ventilators (CoolerMaster: Blade Mater 120) were mounted on a wooden beam at a distance of 40 cm from the devices (Fig. 2a). The ventilators produced wind at a velocity of about 0.2 m/s at a voltage of 0.5 V. At this velocity, the flow of silk threads was in a forward direction. To generate an upward wind we used a ventilator (OK, OTF 802-W) that was fixed on the ground in front of the box (Fig. 2c). This combination of ventilators ensured a wind speed of 0.5–1.5 m/s at the tip of the ballooning units, in line with data on ballooning under natural conditions [42, 43]. The ballooning set-up received artificial light from standard ceiling lamps. Prior to each trial, the following parameters were recorded using a Kestrel 4000 Pocket Weather Meter: the actual wind speed generated (taken at the tip of each ballooning device), room temperature and humidity. Each ballooning unit was used once a day only and was cleaned with ethanol after each trial to remove silk and other possible cues.

Nine days after emergence, the spiderlings (six spiderlings per trial, three trials per family = 18 randomly selected spiderlings) were each carefully transferred to a ballooning unit. After acclimatizing for 1 minute, the ventilators were turned on and the dispersal behaviour was observed during the following 10 min. Spiderlings showed one of two pre-ballooning behaviours at the tip of the skewer: “tip-toe” behaviour or “rappelling”. Tip-toe behaviour entails raising the abdomen, stretching out the legs and simultaneously producing long silk threads by which the spiderling becomes airborne [56]. When rappelling, the spiderling lets itself fall from the tip of the skewer and rides in the air while still attached to the tip with a safety thread. Rappelling spiderlings become airborne by releasing ballooning threads and biting through the safety thread. Since tip-toe behaviour was rare (1.7 %) in agreement with other work on *A. bruennichi* [51, 53], we pooled both behaviours, which are henceforth referred to as ballooning behaviour. Those spiderlings that ballooned were caught in their airborne phase with a sweep net. In this way, 97% of spiderlings (1983 of 2052) could be retrieved. All retrieved spiderlings were weighed on the day of the trial using a high precision balance (Sartorius MES).

### Statistical analyses

Dispersal was the response variable recorded in a binary form (ballooning yes or no). Explanatory variables were of four different kinds: (1) those under the experimenter’s control (reciprocal common garden experiment: winter treatment, origin); (2) inherent to the spiderlings’ body (mass); (3) environmental conditions in the test rooms (wind in m/s, temperature in ^0^C, air pressure in hPa, humidity in %) and (4) uncontrolled experimental conditions (room, observer, time of experiment). All of these variables were included as fixed effects. The spiderlings used in ballooning experiments came from the egg sacs of 114 females (spiderlings from the same egg sac are termed family), that were collected from three populations each from Southern France and the Baltic States. These two variables (family and population) were included as random effects.

We fitted a multiple ANCOVA model to test the effect of the aforementioned potential predictors on ballooning behaviour using a Generalized Linear-Mixed Effect Model (GLMM) with a binomial error structure and logit link. The linear predictor included all explanatory variables mentioned above as additive effects plus the origin:treatment interaction. We assumed a normal distribution for the random variables. To fit the model we used the glmer function from the *lme4 package* [57], which uses Laplace approximation that is suitable for binomial data and a nested design of random effects [58]. Wald tests of fixed effects in the Type II ANODEV table were estimated using a function in the *car package* [59]. Because the air pressure was not recorded for all cases, its effect was tested using a reduced data set. Post-hoc comparisons were based on the treatment contrasts. The statistical significance of effects was assessed from a full model. Statistically significant effects of predictors with less than 5% difference (between factor levels or minimum and maximum predictor value) were, however, considered biologically unimportant. For life-history traits related to reproduction, we examined support for differences in body size and fecundity between females from different origins using a t-test and ANCOVA following Levene’s test on equality of variances. For the ANCOVA, female body size was used as a covariate. Hatching success and offspring survival were compared using Generalized Linear Models (GLM) with Poisson (GLM-p) or binomial (GLM-b) error structures and canonical links. Levene’s test of variance homogeneity was used to compare variances between treatment groups. Descriptive statistics are given as arithmetic means and standard errors of the mean. All analyses were performed with R [60].

## Results

### Dispersal (Fig. 3):

**Fig. 3:**
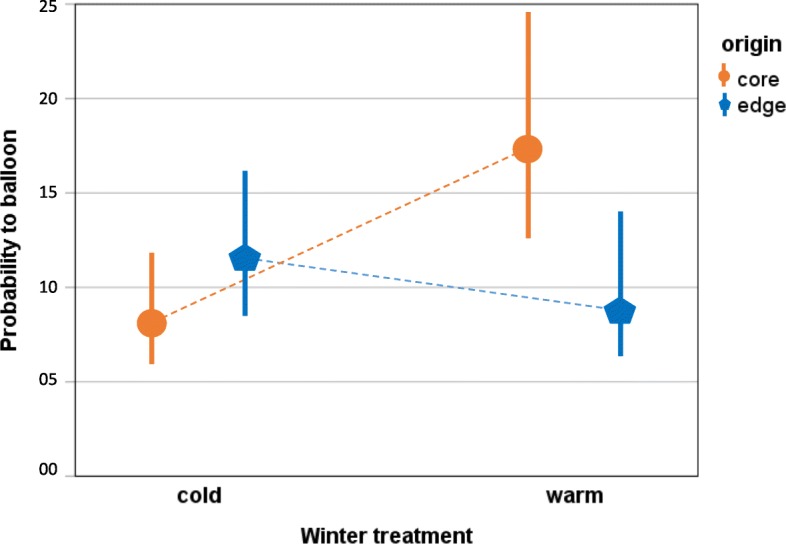
Reciprocal common garden experiment: comparison of the population-average mean probability to balloon (in %, with confidence intervals) for *Argiope bruennichi* spiderlings from the southern core (Southern France) and northern edge (Baltic States) regions after simulated winter conditions for both regions (cold: Baltic States, warm: Southern France). core region: orange circles; edge region: blue pentagons

The fixed effect of origin alone did not significantly explain differences in ballooning activity between the groups (GLMM X^2^_1_ = 0.30, *P* = 0.59). Winter treatment had a marginally significant effect on ballooning probability (GLMM X^2^_1_ = 3.80, *P* = 0.05). The interaction of winter treatment and origin was highly significant (GLMM, X^2^_1_ = 15.2, *P* < 0.0001): after warm winters, spiders from the southern core region had higher probability to balloon (18 %) than after cold winters (8%). On the other hand, spiders from the northern edge region, showed higher ballooning activity after cold winters (12%) compared to warm winters (9%) (Fig. 3). The effect of spiderling body mass on ballooning was not significant (GLMM, *P* > 0.10). Since we tested 18 spiderlings from each family, we also tested for a family effect on ballooning: there was a stronger effect of the family within population ($\hat{\sigma}$ = 1.103) than between populations ($\hat{\sigma}$ = 0.006) (Additional file 1: Figure S2 A,B).

Considering abiotic factors that varied in the test rooms, there was a significant effect of temperature on dispersal activity (GLMM, X^2^_1_ = 6.3, *P* = 0.012): more spiders ballooned when temperatures were higher (Additional file 1: Figure S3). The following variables had a significant effect, but were biologically negligible. Humidity: more spiders ballooned with increasing humidity in the test room (GLMM, X^2^_1_ = 5.9, *P* = 0.014); wind: fewer spiders ballooned with stronger wind (GLMM, X^2^_1_ = 7.5, *P* = 0.006); air pressure: fewer spiders ballooned with higher air pressure in the test room (GLMM, X^2^_1_ = 858.3, *P* < 0.0001). The effect of time of day at testing was not significant (GLMM, X^2^_1_ = 0.2, *P* = 0.65). Descriptive statistics of the environmental conditions in the test rooms are given in additional file 1: Table S4.

### Life-history traits related to reproduction (Fig. 4)

**Fig. 4:**
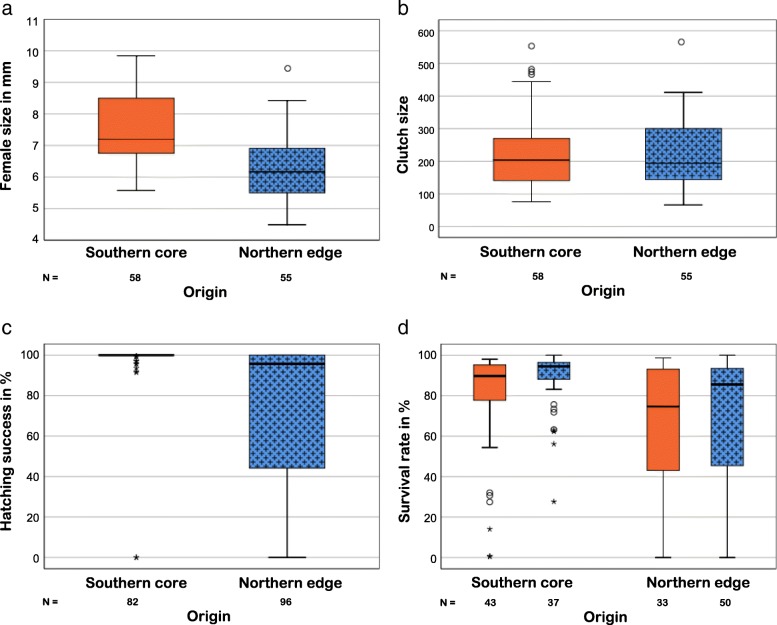
Life-history traits of female *Argiope bruennichi* from core and edge populations. **a** Differences in body size measured as length of patella + tibia (in mm) for the first legs (*N* = 113). **b** Clutch size measured as total number of eggs oviposited in an egg sac (N = 113). **c** Hatching success measured as number of offspring hatched divided by the total number of eggs laid (*N* = 178). **d** Survival probability of spiderlings (measured as number of offspring alive after winter treatment divided by the number of offspring hatched) depending on origin and winter conditions (*N* = 163). Southern core populations are depicted in orange, northern edge populations in blue, frecked pattern. Thick lines indicate medians, boxes represent quartiles and whiskers show 1.5 times the interquartile range. Open circles depict outliers and asterisks extreme outliers (> 3.0 times IR)

Females collected from the edge of the distribution differed in body size from those from the core. Females from the northern edge populations were significantly smaller (6.33 ± 1.43 mm) than those from the southern core (7.52 ± 1.34 mm) (Fig. 4a) (t-test: t_111_ = − 6.06; *P* < 0.001). Females from the northern edge populations laid a similar number of eggs (226.04 ± 14.68) as those from the core of the distribution (228.43 ± 14.75) (Fig. 4b) (t-test: t_111_ = − 0.06; *P* = 0.954). Consequently, females from the northern edge invested significantly more into reproduction than females from the core region relative to body size (ANCOVA: F_1,109_ = 6.56, *P* = 0.012). Average offspring weight per family did not differ between core and edge (Southern France: 0.383 ± 0.0046 mg; Baltic States: 0.379 ± 0.0076 mg; ANCOVA: F_1,110_ = 0.002, *P* = 0.967, *N* = 113) and was not related to the size of the mother (ANCOVA: F_1,110_ = 0.40, *P* = 0.530).

Hatching of spiderlings occurred under simulated native conditions before the winter treatments started. Interestingly, hatching success was very high and hardly variable in egg sacs from the southern core (97.08 %, *N* = 82) but was significantly lower and highly variable in egg sacs from the edge populations (73.27 %, *N* = 96) (GLM-b: X^2^_1_ = 27.41, *P* < 0.001) (Fig. 4c). Egg sacs from the northern edge had a significantly higher probability of hatching failure (13 of 96 egg sacs from the northern edge; 2 of 82 from the southern core; GLM-b: X^2^_1_ = 7.06, *P* = 0.008). For those egg sacs with hatching success (163 of 178, 91.6 %), the survival rate of spiderlings after the winter treatments was recorded. Survival rate was significantly higher for spiderlings from the southern core (82.78 %, *N* = 80) than for spiderlings from the northern edge (66.15 %, *N* = 83) (GLM-b: X^2^_1_ = 13.57, P < 0.001) (Fig. 4d). The winter treatment did not have a significant effect on winter survival (GLM-b: X^2^_1_ = 1.20, *P* = 0.158), nor was the interaction between winter treatment and origin significant (GLM-b: X^2^_1_ = 0.25, *P* = 0.621). Interestingly, the variance in survival was significantly higher in egg sacs produced by females from the northern edge relative to those from the southern core region, irrespective of winter treatment (Levene test: t_161_ = − 3.54, *P* = 0.001). Overall, no relationship between mean dispersal per family and fecundity of the mother (GLM-p, X^2^_1_ = 0.60, *P* = 0.807) or survival rate (GLM-p: X^2^_1_ = 2.63, *P* = 0.608) was found.

## Discussion

Spiderlings of *A. bruennichi* from the northern edge populations are not more prone to disperse than those from the southern core. The greatest differentiation found was for core populations relating to winter conditions. Even under matching winter conditions dispersal in offspring was lower in northern edge populations relative to offspring from core populations. Hence, we found no evidence for ecologically relevant higher dispersal in northern range populations as predicted by spatial sorting. Dispersal responses were, however, related to the winter conditions in a relative, and not absolute, sense as spiderlings show increased ballooning after experiencing their native winter conditions and showed reduced dispersal under non-matching winter conditions. As spiderlings of orb-weaving spiders typically show a clumped distribution close to the egg sac for several days post emergence, increased dispersal after matching winter conditions might be interpreted as an adaptive response to anticipated higher densities, and hence (kin)-competition [34, 37]. Conversely, stressful conditions related to non-matching thermal winter conditions may carry-over to behaviour in later life stages [61] and constrain dispersal. As we did not find differences in winter survival in response to these putative mismatches, we suggest that the dispersal rate more likely reflects the anticipated degree of competition. We additionally found a strong family effect on ballooning propensity that was larger than the difference between populations. This suggests that ballooning is under strong maternal control by intergenerational plasticity, rather than by genetic effects.

Theory predicts higher dispersal activity at the edges of expanding populations through spatial sorting [17, 19]. However, we found no evidence for spatial sorting in the range expanding *A. bruennichi*. Spatial sorting patterns may become obscured when local selection against dispersal is severe [62, 63]. Range margins rarely offer large quantities of suitable habitats but instead, highly fragmented, distant patches, which seems to be the case for *A. bruennichi* in the Baltic States where there are large areas of forest between suitable patches (Uhl G, unpublished). In small founder populations, the selective advantage of dispersal might be reduced due to lower chances of survival beyond the current range and reproductive trade-offs with dispersal [64]. Further, reproduction in marginal habitats can be inhibited and the costs of finding a mate in low-density populations can be severe [65]. Allee effects with positive density-dependent individual fitness in sparse populations can limit dispersal propensity [64]. Consequently, dispersal rates can return quickly to that of the core population after the initial stage of colonization [66, 67]. Such transient changes in dispersal were e.g. demonstrated for wing-dimorphic bush crickets that show a high frequency of long-winged individuals in recently colonized habitats but reversions to frequencies similar to the core after just 5–10 years of colonization [68]. It is also possible that our populations might not represent the very leading edge and that the assumed higher dispersal rate during the initial stages of colonization has already reverted to lower dispersal rates [67]. The age of our populations is not precisely known but is very likely to be around 5–10 years (see information on records given in Material and Methods). If these records mirror the distribution and range expansion of *A. bruennichi* adequately, it can be assumed that its dispersal behaviour reverted to lower frequencies within a similarly short time as in bush crickets [68].

According to theory, life-history traits are under very different selection pressures at the range edge compared to the core. We found that females are significantly smaller at the edge relative to the core. Since the number of moulting stages in spiders can be variable [69], the smaller size suggests that spiders from the Baltic States omit at least one molting stage to adulthood thereby shortening developmental time. Advancing the beginning of the reproductive season ensures a match between adulthood and suitable environmental conditions for reproduction [70, 71], which can explain why mating and egg production occur almost concurrently between the two regions. Similar shifts in the timing of maturity have been demonstrated to be highly adaptive in range expanding arthropods [26].

Spiders from edge populations are as fecund as those from core populations, and hence have a higher number of offspring relative to their body size. Since total offspring biomass did not differ between regions, we conclude that *A. bruennichi* individuals from the northern edge invest more in reproduction than individuals from the core population. This is in line with the theoretical prediction that populations at the expanding front are defined by density-independent population growth, and thereby experience an r- selective environment relative to the individuals in a core population [19, 24]. Higher investment in reproduction relative to other traits such as growth has been found in range expanding butterflies [72, 73]. The higher relative reproductive rate might also arise from enemy release effects, i.e. reduced pressure from natural enemies, including not only predators, but also pathogens and parasites [74, 75]. Indeed, it was shown that introduced animals are, on average, less predated on and infected by significantly fewer parasite species than in their native range [76]. Predation by other spiders and birds on adult *Argiope* and their egg sacs as well as egg parasitism is common [77, 78]. Consequently, a lower total abundance of predators, specialist parasites and parasitoids might cause an enemy release effect at higher latitudes that allows females to increase investment in reproduction.

Fecundity assessment alone would not provide sufficient insight into the costs and benefits of range expansion as is demonstrated by our data on origin-dependent differential survival in *A. bruennichi*. Survival in the egg sacs from France was high irrespective of the winter treatment, demonstrating that spiderlings from core populations can survive adverse winter conditions. Spiderlings from the expanding edge, however, had a significantly lower survival probability overall, irrespective of the winter conditions they experienced. Furthermore, variance in survival was extremely high in egg sacs from the northern edge populations compared to those from the core. This unexpected finding might relate to variance in maternal condition that affects the provisioning of spiderlings with essential resources. However, spiderlings from edge populations did not differ in weight nor was the variance in female body size and clutch size higher in edge populations compared to core populations (Fig. 4a, b). Higher survival variance in edge populations might result from local inbreeding in line with the findings that many species show reduced genetic diversity towards their latitudinal range margins [13]. The pattern in *A. bruennichi*, however, seems to be more complex as overall higher level of genetic diversity was found in the northern region compared to the core region [45]. An explanation for this possible discrepancy might be that populations in the north are small, isolated and suffer from inbreeding, and are genetically differentiated from other populations. Furthermore, the more diverse northern populations were shown to share alleles with populations from the East of the Palearctic distribution [79, 80]. This highly interesting pattern should be revisited as it may reveal stochastic effects combined with signatures of inbreeding and admixture.

## Conclusions

We show that there are no systematic differences in dispersal between core and edge populations that would indicate relevant spatial sorting during range expansion in *A. bruennichi*. Dispersal behaviour between core and recently colonised edge populations is highly variable and spiders showed highest dispersal activity after having experienced matching winter conditions. Dispersal rates were found to be conditional on earlier experiences of winter climate, under maternal control, and not correlated to traits related to reproduction and survival. Reproductive investment was found to be higher in the northern edge populations, suggesting that reproduction is decoupled from dispersal, thereby adding support for dispersal evolving independently from other vital traits within species [30].

## Supplementary information

**Additional file 1: Table S1.** Coordinates of collecting sites of *Argiope bruennichi* in the core region of Southern France and the range edge region of the Baltic States. **Figure S1.** Temperature regimes of collecting sites (see Table S1) and climate chambers in which winter simulations took place. **Table S2.** Average day and night temperatures in the climate cabinets simulating temperature conditions of the populations from Southern France and Baltic States over the course of the reciprocal common garden experiment. **Table S3.** Sample sizes for the reciprocal common garden experiment. **Table S4.** Descriptive statistics for environmental conditions in the test room during the ballooning trials. **Figure S2.** Comparison (boxplot) of probabilities to balloon (%) for (**A**) the offspring of the females collected and (**B**) populations of *Argiope bruennichi* from Baltic States and Southern France. **Figure S3.** Relationship between the probability of a spiderling to balloon and the temperature in the test room. Estimated logit model is shown.

## Data Availability

The datasets used and/or analysed during the current study are available from the corresponding author on request.
