# Supplementary material for: Dispersal and life-history traits in a spider with rapid range expansion
Source: Mov Ecol. 2020 Jan 7;8:2. doi: 10.1186/s40462-019-0182-4 (PMC6947977; doi:10.1186/s40462-019-0182-4)
Supplement: Supplementary file 1 — Additional file 1: Table S1. Coordinates of collecting sites of Argiope bruennichi in the core region of Southern France and the range edge region of the Baltic States. Figure S1. Temperature regimes of collecting sites (see Table S1) and climate chambers in which winter simulations took place. Table S2. Average day and night temperatures in the climate cabinets simulating temperature conditions of the populations from Southern France and Baltic States over the course of the reciprocal common garden experiment. Table S3. Sample sizes for the reciprocal common garden experiment. Table S4. Descriptive statistics for environmental conditions in the test room during the ballooning trials. Figure S2. Comparison (boxplot) of probabilities to balloon (%) for (A) the offspring of the females collected and (B) populations of Argiope bruennichi from Baltic States and Southern France. Figure S3. Relationship between the probability of a spiderling to balloon and the temperature in the test room. Estimated logit model is shown. [file 40462_2019_182_MOESM1_ESM.pdf]

Supplementary information

Dispersal and life-history traits in a spider with rapid range expansion

Marina Wolz; Michael Klockmann; Torben Schmitz; Stano Pekár; Dries Bonte & Gabriele Uhl

gabriele.uhl@uni-greifswald.de

Table S1. Coordinates of collecting sites of *Argiope bruennichi* in the core region of Southern France and the range edge region of the Baltic States

**Origin**	**Population**	**Coordinates**
**Southern France**		
	Belflou	43°19’23.60”N; 1°47`13.74”E
	Casties near Cazalrenoux	43°11`12.18”N; 1°55`41.40”E
	Domaine de Perry, Pieusse	43°03`57.76”N; 2°16`39.97”E
**Baltic States**		
	Virtsu, Estonia	58°33'34.7"N; 23°33'06.5"E
	Pärnu, Estonia	58°20'04.2"N; 24°35'05.0"E
	Ainaži, Latvia	57°50'49.5"N; 24°20'57.5"E

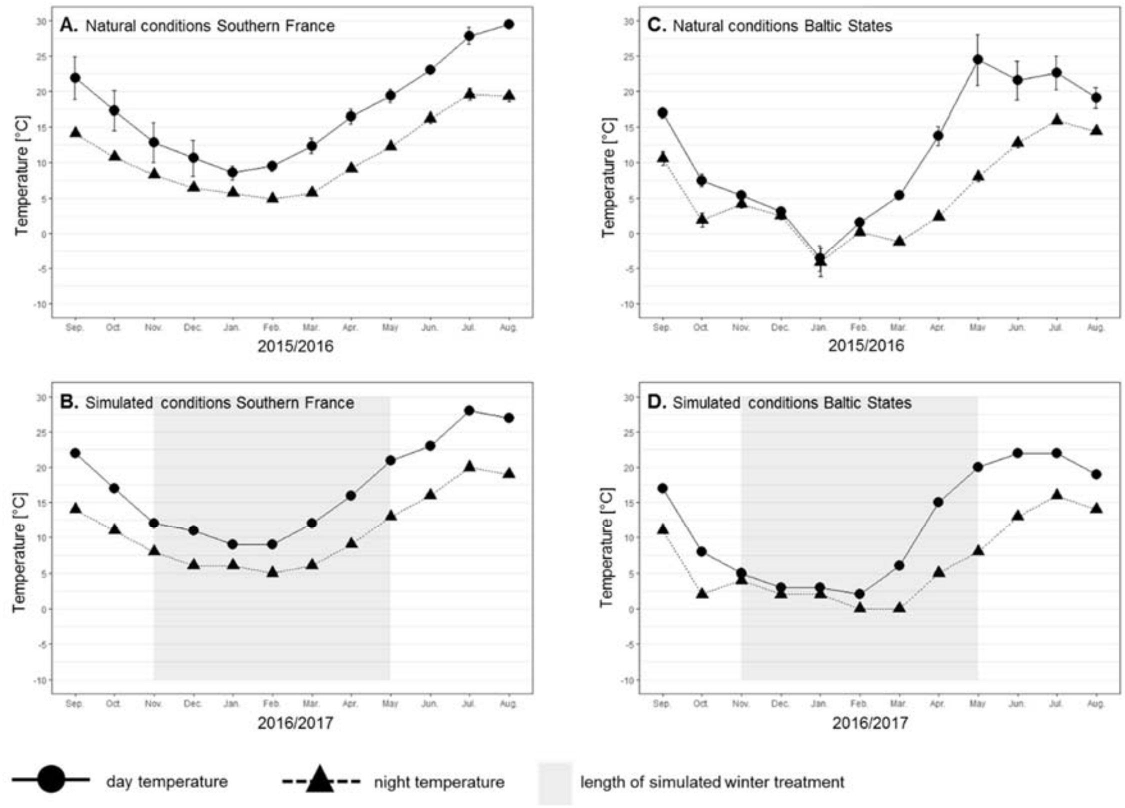

Figure S1: Temperature regimes of collecting sites (see Table S1) and climate chambers in which winter simulations took place. Black dots represent day temperatures, black triangles represent night temperature. A, C: Mean natural day and night air temperature at the two collecting sites (A: Southern France and C: Baltic States) as recorded by data loggers from Sept 2015 to August 2016 (error bars represent monthly mean temperatures). B, D: Simulated conditions in climate chambers and cabinets to which females and subsequently spiderlings in egg sacs were subject to in the laboratory. The grey shaded areas indicates the length of the winter treatments 2016/2017 for spiderlings in the egg sacs before the ballooning trials. A summary of the applied frost days, ice days and freeze-thaw days is given in Table S2.

Table S2: Average day and night temperatures in the climate cabinets simulating temperature conditions of the populations from Southern France and Baltic States over the course of the reciprocal common garden experiment. “Freeze-thaw days” are days with night temperatures of -2°C and day temperatures above 0°C (12:12 hours). “Ice days” are days with -2°C for 24 hours. The grey shaded areas represent the conditions the egg sacs were subject to during winter treatments. Temperature regimes were simulated according to data logger information from the previous year at the collecting sites.

	Simulated conditions Southern France			Simulated conditions Baltic States			
month	day temperature [C°]	night temperature [C°]	freeze-thaw days [N]	day temperature [C°]	night temperature [C°]	freeze-thaw days [N]	ice days [N]
October	17	11		8	2		
November	12	8	1	5	4	2	2
December	11	6		3	2	2	6
January	9	6	3	3	2	1	26
February	9	5	2	2	0	11	1
March	12	6		6	0	23	2
April	16	9		15	5	10	
May	21	13		20	8	1	
June	23	16		22	13		
July	28	20		22	16		
August	27	19		19	14		
September	22	14		17	11		

Table S3: Sample sizes for the reciprocal common garden experiment. Three replicate populations were sampled from Southern France and the Baltic States (for coordinates see Tab. S1). In winter treatments, thermal conditions from both regions were simulated according to data from data loggers (see Fig. S1). The number of egg sacs and the number of spiderlings (individuals) tested per population are given. 18 spiderlings were tested per egg sac. A total of 114 egg sacs were investigated.

		Southern France Populations				Baltic State Populations			
winter treatment	sample size (N)	Belflou	Casties	Pieusse	total number	Virtsu	Pärnu	Ainaži	total number
Southern France	egg sacs	9	10	10	29	9	10	8	27
	individuals	162	180	180	522	162	180	144	486
Baltic States	egg sacs	9	10	10	29	10	10	9	29
	individuals	162	180	180	522	180	180	162	522

Table S4. Descriptive statistics for environmental conditions in the test room during the ballooning trials.

	N	minimum	maximum	mean	standard error
Temperature ^{0}C	114	19.30	25.40	22.81	.109
Humidity %	114	16.70	62.80	37.97	.849
Wind speed m/s	114	.50	1.60	.80	.019
Air pressure hPa	48	999.90	1024.50	1014.05	.953

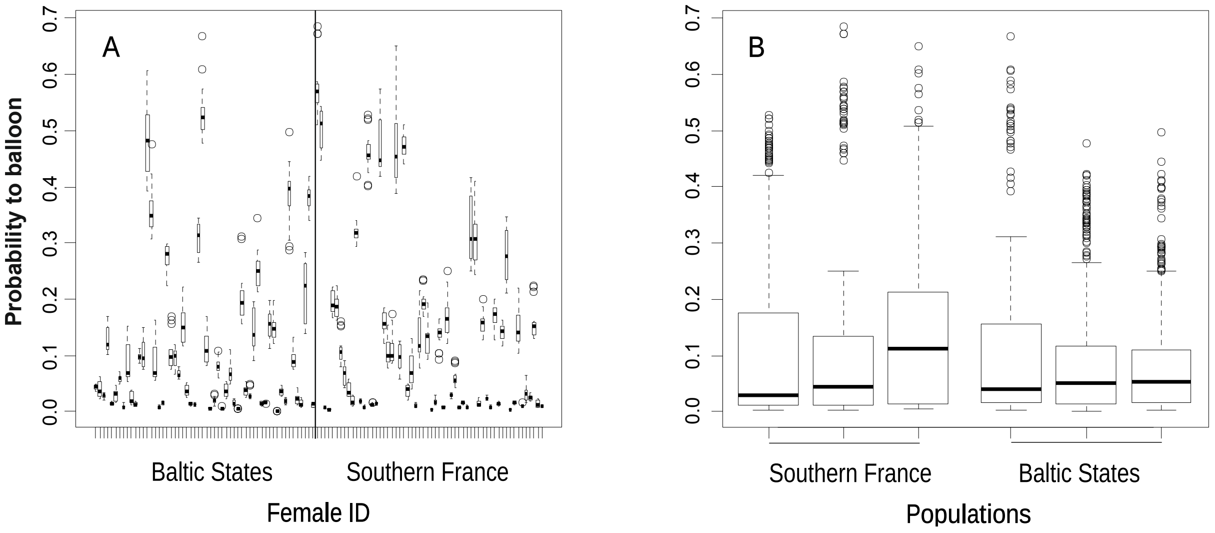

Figure S2. Comparison (boxplot) of probabilities to balloon (%) for (**A**) offspring of the females collected and (**B**) populations of *Argiope bruennichi* from Baltic States and Southern France. Thick lines are medians, boxes are quartiles and whiskers are 1.5 times interquartile range. Open circles depict outliers.

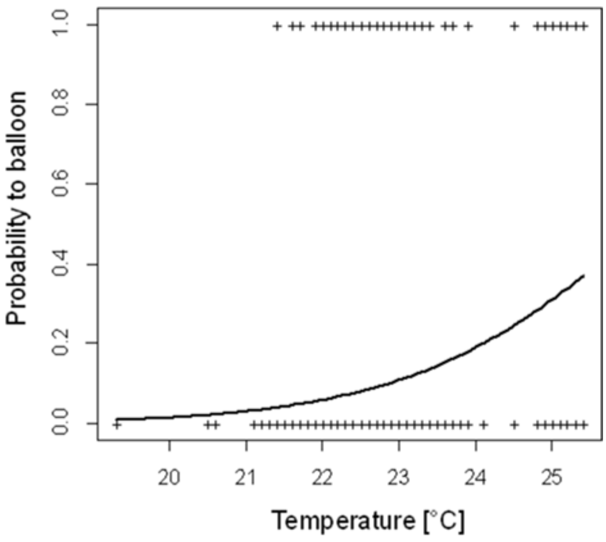

Figure S3. Relationship between the probability of a spiderling to balloon and the temperature in the test room. Estimated logit model is shown.
